# Comparison of oesophageal function tests between Chinese non-erosive reflux disease and reflux hypersensitivity patients

**DOI:** 10.1186/s12876-017-0624-7

**Published:** 2017-05-23

**Authors:** Feng Gao, Yan Gao, Xue Chen, Jie Qian, Jie Zhang

**Affiliations:** 1 Digestive Department, Beijing Anzhen Hospital,Capital Medical University, Beijing, 100029 China; 2 Digestive Department, Beijing Chao-Yang Hospital,Capital Medical University, Beijing, 100020 China

**Keywords:** High-resolution manometry and impedance, Non-erosive reflux disease, 24-h multi-channel intraluminal impedance and pH recording

## Abstract

**Background:**

By means of 24 h multi-channel intraluminal impedance and pH recording (MII/pH), patients with heartburn and normal upper gastrointestinal endoscopy findings can be classified into those with non-erosive reflux disease (NERD) and those with reflux hypersensitivity (RH). Therefore, in this study, we investigated the difference in oesophageal function tests in Chinese patients with NERD and RH.

**Methods:**

NERD patients were selected from the digestive department, Beijing Anzhen Hospital and Beijing Chao-Yang Hospital, Capital Medical University, after upper gastrointestinal endoscope, high-resolution manometry and impedance (HRiM), and MII/pH examinations between 2014 and 2016.

**Results:**

In total, 111 NERD patients with abnormal acid exposure, and 92 RH patients were enrolled. Values for NERD and RH were as follows: lower oesophageal sphincter pressure, 15.3 ± 8.9 and 19.3 ± 23.3 mmHg (*P* = 0.122); integrated relaxation pressure, 7.5 ± 4.8 and 7.9 ± 5.2 mmHg (*P* = 0.485); distal contractile integral, 751.9 ± 856.2 and 661.9 ± 961.7 mmHg∙s∙cm (*P* = 0.482); ineffective oesophageal motility rate, 49.5% and 41.3% (*P* = 0.241); fragmented peristalsis rate, 5.4% and 9.8% (*P* = 0.235); hiatal hernia rate, 9.0% and 8.6% (*P* = 0.938); total bolus transit time, 6.3 ± 1.3 and 6.5 ± 1.3 s (*P* = 0.119); complete bolus transit rate, 76.1 ± 33.0% and 73.1 ± 32.0% (*P* = 0.224); total acid exposure time, 6.1 ± 3.7% and 0.8 ± 0.8% (*P <* 0.001); total bolus exposure time, 2.5 ± 2.1% and 1.5 ± 1.1% (*P <* 0.001); proximal acid reflux events, 13.2 ± 10.5 and 9.7 ± 8.9 (*P* = 0.011); distal acid reflux events, 25.3 ± 15.8 and 13.4 ± 11.2 (*P <* 0.001); post-reflux swallow-induced peristaltic wave index, 25.1 ± 9.5% and 32.6 ± 15.2% (*P* < 0.001); and mean nocturnal baseline impedance, 1,450.2 ± 750.8 and 2,503.6 ± 964.1 ohms (*P <* 0.001), respectively.

**Conclusions:**

NERD and RH patients showed similar values on HRiM. NERD patients had greater acid exposure time, bolus exposure time, proximal and distal acid reflux events, and increased impairment of chemical clearance and mucosal integrity than RH patients. NERD and RH should be classified correctly by MII/pH to provide adequate relief from related symptoms.

## Background

Non-erosive reflux disease (NERD) is characterised by the absence of oesophageal mucosal damage during upper gastrointestinal endoscopy, despite the presence of the ‘classic’ symptoms of gastroesophageal reflux, such as heartburn and acid reflux [1]. Additionally, subjects with heartburn and normal upper gastrointestinal endoscopy represent a heterogeneous group of patients, some of which may not actually have gastro-oesophageal reflux-related disorders [2–4]. With the clinical application of 24-h multi-channel intraluminal impedance and pH recording (MII/pH), patients with heartburn and normal upper gastrointestinal endoscopic examination can be classified into NERD (with abnormal acid exposure, positive or negative symptom reflux association), reflux hypersensitivity (RH, normal acid exposure with positive symptom reflux association), and functional heartburn (FH, normal acid exposure with negative symptom reflux association) [3–5]. RH identifies patients with oesophageal symptoms (heartburn or chest pain) that would be considered within the gastroesophageal reflux disease (GERD) realm on clinical presentation, without reflux evidence on endoscopy or pH-impedance monitoring, but with demonstration of triggering of symptoms by physiological reflux [5]. However, few studies have assessed differences between NERD and RH patients [6]. Thus, we investigated differences in oesophageal function tests in a Chinese population with NERD and RH.

## Methods

### Ethics

The study received ethics approval from the local ethics board of Beijing Anzhen Hospital, Capital Medical University. All participants gave written informed consent.

### Patient selection

Chinese patients who presented with a main symptom of heartburn with a normal upper gastrointestinal endoscopic examination, and underwent high-resolution manometry and impedance (HRiM), and 24-h multi-channel intraluminal impedance and pH recording (MII/pH) in the digestive department of Beijing Anzhen Hospital and Beijing Chao-Yang Hospital between December 2014 and December 2016 were enrolled. Patients with other chronic active medical diseases (such as coronary artery disease, hypertension, malignancy, and diabetes mellitus) were excluded.

Patients with heartburn and a normal upper gastrointestinal endoscope exam, abnormal oesophageal acid exposure, and positive or negative symptom reflux association, were diagnosed with NERD. The diagnostic criteria for RH were all of the following: (i) the presence of retrosternal symptoms, including heartburn and chest pain, (ii) normal endoscopy and absence of evidence that eosinophilic o oesophagitis was the cause of the symptoms, (iii) absence of major oesophageal motor disorders (achalasia/oesophagogastric junction (EGJ) outflow obstruction, diffuse oesophageal spasm, jackhammer oesophagus, and absent peristalsis), and (iv) evidence of triggering of symptoms by reflux events despite normal acid exposure on pH or pH-impedance monitoring (response to anti-secretory therapy does not exclude the diagnosis). The criteria had to be fulfilled for 3 months prior to the beginning of the study, with symptom onset at least 6 months prior to diagnosis with a frequency of at least twice per week [3–5].

### High-resolution oesophageal manometry and impedance

A specially designed solid-state manometry catheter (Sandhill Scientific Inc., Highland Ranch, CO, USA) with 32 manometric sensors and four pairs of MII sensors, separated by 5-cm intervals, was used to assess oesophageal pressures and impedance with the patient in a supine position. The lower oesophageal sphincter (LES) was examined with distal circumferential manometric sensors. The catheter was positioned so that the pressure transducers were located across the upper oesophageal sphincter, oesophageal body, and LES, and the distal channels were in the stomach. Ten swallows with 5 mL normal (0.9%) saline solution were then performed at 30-s intervals.

### 24-h Oesophageal multi-channel intraluminal impedance and pH recordings

The 2.1-mm outer diameter study catheter consisted of six electrode pairs for measuring intraluminal impedance (3, 5, 7, 9, 15, and 17 cm above the LES) and an antimony pH sensor 5 cm above the LES (Sandhill Scientific Inc., Highland Ranch, CO, USA). An impedance amplifier delivered an ultra-low current in a range of 1-2 kHz, with resulting current flow variations in response to intraluminal impedance changes (high impedance indicates gas or air; low impedance indicates liquid). Signals from the six impedance channels and the pH channel were recorded at 50 samples per second. The data were stored in an ambulatory recorder and saved on a 256-MB CompactFlash card. Event markers recorded the occurrence of symptoms, times of meals, and changes in body position. The study was performed on an outpatient basis after an overnight fast with the LES located by oesophageal manometry. The patients undertook HRiM and MII/pH with a 7-day washout for proton pump inhibitors and/or H_2_ antagonists.

### Data collection

Oesophageal bolus clearance can be assessed by measurement of total bolus transit time by classifying swallows as complete bolus transit (if bolus entry occurs at the most proximal site and bolus exit points are recorded in all three distal recording segments) or as incomplete bolus transit (if bolus exit is not identified at any of the three distal recording segments), and in terms of the complete bolus transit rate. The distal contractile integral (DCI) of the distal segmental contraction is a parameter that integrates contractile pressure (mmHg), duration (s) of contraction, and the length of the smooth muscle oesophagus (cm). Distal oesophageal amplitude (DEA) is an average of the contraction amplitude at 5 and 10 cm above the LES. Integrated relaxation pressure (IRP) reports the mean EGJ pressure, which is measured with an electronic equivalent of a sleeve sensor for four continuous or non-continuous seconds of relaxation in a 10-s window following deglutitive UES relaxation. The parameters of the length of the lower oesophageal sphincter (LESL), the lower oesophageal sphincter pressure (LESP), the lower oesophageal sphincter residual pressure (LESRP), and the upper oesophageal sphincter pressure (UESP) were also measured [7, 8]. Ineffective oesophageal motility (IEM) was defined as at least 50% of swallows with a DCI below 450 mmHg∙s∙cm [9]. Fragmented peristalsis was defined as at least 50% of fragmented swallows (contractions with DCI > 450 mmHg∙s∙cm and break > 5 cm in the 20 mmHg isobaric contour) [9]. The parameters of DeMeester score, acid exposure upright (%), acid exposure recumbent (%), acid exposure total (%), bolus exposure upright (%), bolus exposure recumbent (%), bolus exposure total (%), proximal acid events, proximal nonacid events, proximal total events, distal acid reflux events, distal non-acid reflux events, and distal total reflux events were measured [10]. Symptoms were considered to be associated with reflux if they occurred within a 2-min window after the onset of the reflux episode [10]. The symptom index (SI) was considered positive if the value was 50% or more; symptom association probability (SAP) was considered positive if it was 95% or more [11, 12]. All parameters were measured using Bio View Analysis software (Sandhill Scientific, Inc., Highland Ranch, CO, USA).

A post-reflux swallow-induced peristaltic wave (PSPW) was defined as an antegrade 50% drop in impedance relative to the pre-swallow baseline originating in the most-proximal impedance site, reaching all distal impedance sites, and followed by a return to at least 50% of the baseline in the distal impedance sites (bolus exit). Post-reflux swallows that did not reach the distal impedance sites, or that occurred more than 30 s after the end of reflux episodes, were not considered. For each impedance-pH tracing, the number of refluxes followed within 30 s by a PSPW was divided by the number of total refluxes (manual calculation) to obtain the PSPW index [13].

Mean nocturnal baseline impedance (MNBI) was assessed from the most distal impedance channel during a night recumbency period. Three 10-min time periods (at around 1:00 AM, 2:00 AM, and 3:00 AM) were selected, and the mean baseline for each period was computed with the aid of the software. Time periods including swallows, refluxes, and pH drops were avoided. The mean of the three measurements was manually calculated to obtain the MNBI [14].

### Comparison groups

There were two groups in the study: Chinese NERD patients and RH patients. The diagnosis of acid exposure was according to the results of MII/pH, presenting an abnormal upright acid exposure time (≥6.3%), recumbent acid exposure time (≥1.2%), or total acid exposure time (≥4.2%) [10].

### Statistical methods

Categorical data are described as means ± standard deviation (SD). Data were analysed using the independent-samples *t*-test or *χ* ^2^ test. A p-value < 0.05 was considered to indicate statistical significance. All data were analysed with the SPSS software (ver. 17.0, IBM Corp., Armonk, NY, USA).

## Results

In total, 351 patients with heartburn for at least 6 months with a frequency of at least twice a week and without (or with ineffective) PPI treatment, and normal upper gastrointestinal endoscope examinations underwent HRM/Z and MII/pH testing between December 2013 and December 2015, 148 of whom with normal acid exposure and negative SAP and diagnosed with functional heartburn were excluded. Also, 111 patients with abnormal acid exposure were diagnosed with NERD, and 92 patients with normal acid exposure and positive SAP were diagnosed with RH; 20 patients had a history of ineffective PPI treatment (12 in the NERD group and 8 in the RH group). The group with NERD was significantly older than the group with RH. The groups showed no difference in gender.

The HRiM results between NERD and RH patients are shown in Table 1. The NERD and RH groups had similar values for LESP, LESRP, IRP, UESP, DEA, DCI, total bolus transit time, and complete bolus transit rates. The NERD and RH groups also had similar rates of IEM, fragmented peristalsis, and hiatal hernia.

**Table 1:** Demographic data and high-resolution manometry and impedance results

Items	NERD	RH	Independent-sample *t*-test or *χ* ^2^ test
	*n* = 111	*n* = 92	
Age (mean ± SD, years)	58.8 ± 10.4	51.1 ± 12.8	*P* < 0.001
Male/Female, *n*	51/60	44/48	*P* = 0.789
LESP (mean ± SD, mmHg)	15.3 ± 8.9	19.3 ± 23.3	*P* = 0.122
LESL (mean ± SD, cm)	3.9 ± 1.6	3.7 ± 0.7	*P* = 0.343
LESRP (mean ± SD, mmHg)	4.3 ± 4.0	4.6 ± 4.3	*P* = 0.564
IRP (mean ± SD, mmHg)	7.5 ± 4.8	7.9 ± 5.2	*P* = 0.485
UESP (mean ± SD, mmHg)	73.2 ± 29.6	82.1 ± 35.9	*P* = 0.054
DEA (mean ± SD, mmHg)	65.5 ± 36.7	64.0 ± 32.9	*P* = 0.756
DCI (mean ± SD, mmHg∙s∙cm)	751.9 ± 856.2	661.9 ± 961.7	*P* = 0.482
Ineffective oesophageal motility *n* (%)	55(49.5)	38(41.3)	*P* = 0.241
Fragmented peristalsis %	6(5.4)	9(9.8)	*P* = 0.235
Total bolus transit time (s)	6.3 ± 1.3	6.5 ± 1.3	*P* = 0.119
Complete bolus transit rate (%)	76.1 ± 33.0	73.1 ± 32.0	*P* = 0.224
Hiatus hernia n(%)	10(9.0)	8(8.6)	*P* = 0.938

*NERD* non-erosive reflux disease, *RH* reflux hypersensitivity, *LESP* lower oesophageal sphincter pressure, *LESL* length of lower oesophageal sphincter, *LESRP* lower oesophageal sphincter residual pressure, *IRP* integrated relaxation pressure, *UESP* upper oesophageal sphincter pressure, *DEA* distal oesophageal amplitude, *DCI* distal contractile integral, *SD* standard deviation

The MII/pH results between NERD and RH patients are shown in Table 2. Compared with RH patients, NERD patients had significantly higher values for DeMeester score, acid exposure upright time, acid exposure recumbent time, acid exposure total time, bolus exposure upright time, bolus exposure recumbent time, and bolus exposure total time. Compared with RH patients, NERD patients showed significantly more proximal and distal acid reflux events. NERD patients had significantly fewer distal nonacid reflux events. NERD patients also had significantly lower PSPW index and MNBI values.

**Table 2:** Results of 24-h multi-channel intraluminal impedance and pH recording

Items	NERD	RH	Independent-sample *t*-test or χ^2^test
	*n* = 111	*n* = 92	
DeMeester	23.4 ± 13.0	4.0 ± 6.6	*P < 0.001*
Acid exposure upright (%)	7.7 ± 6.1	1.4 ± 1.4	*P < 0.001*
Acid exposure recumbent (%)	4.8 ± 5.0	0.1 ± 0.2	*P < 0.001*
Acid exposure total (%)	6.1 ± 3.7	0.8 ± 0.8	*P < 0.001*
Bolus exposure upright (%)	3.9 ± 2.8	2.6 ± 2.0	*P < 0.001*
Bolus exposure recumbent (%)	1.2 ± 1.9	0.4 ± 0.7	*P < 0.001*
Bolus exposure total (%)	2.5 ± 2.1	1.5 ± 1.1	*P < 0.001*
Proximal acid event (*n*)	13.2 ± 10.5	9.7 ± 8.9	*P* = 0.011
Proximal nonacid event (*n*)	10.1 ± 9.2	11.6 ± 10.7	*P* = 0.284
Proximal total event (*n*)	23.4 ± 15.4	20.8 ± 16.8	*P* = 0.258
Distal acid reflux event (*n*)	25.3 ± 15.8	13.4 ± 11.2	*P < 0.001*
Distal nonacid reflux event (*n*)	19.5 ± 14.9	25.1 ± 15.2	*P* = 0.009
Distal total reflux event (*n*)	44.8 ± 24.7	38.5 ± 20.7	*P* = 0.051
PSPW index (%)	25.1 ± 9.5	32.6 ± 15.2	*P < 0.001*
MNBI (ohms)	1450.2 ± 750.8	2503.6 ± 964.1	*P < 0.001*

*NERD* non-erosive reflux disease, *RH* reflux hypersensitivity PSPW index, post-reflux swallow-induced peristaltic wave index, *MNBI* mean nocturnal baseline impedance

## Discussion

The Vevey Consensus Group defined NERD as a subcategory of GERD, characterised by troublesome reflux-related symptoms in the absence of oesophageal erosions/breaks on conventional endoscopy and without recent acid-suppressive therapy [15]. Compared with erosive oesophagitis patients, NERD patients appear to be less responsive to proton pump inhibitors (PPIs) [16] and have a lower hiatal hernia rate and oesophageal dysmotility [17]. The prevalence of NERD is estimated to be about 50–70% of the GERD population in Western countries [18, 19]. In Asia, NERD is reported to differentially affect different ethnic GERD populations, such as 60–90% of Chinese, 65% of Indians, and 72% of Malays [20]. With the advent of impedance studies [21, 22], NERD patients have been shown to have less total acid and weak acid reflux [23], and to be more sensitive to weak acid reflux than erosive oesophagitis patients. Proximal migration of acid and non-acid reflux seems to play a role in symptom generation in NERD [24]. RH identifies patients with oesophageal symptoms (heartburn or chest pain) that would be considered within the GERD realm on clinical presentation, without reflux evidence on endoscopy or pH-impedance monitoring, but with demonstration of triggering of symptoms by physiological reflux [5].

In this study, we compared oesophageal HRiM and MII/pH values between NERD and RH patients with the same Sandhill system and in the supine position. NERD and RH patients showed similar values of LESP, LESL, LESRP, IRP, UESP, DEA, and DCI. It is known that the primary determinant of GERD severity is a dysfunctional anti-reflux barrier and impaired oesophageal clearance. Anti-reflux barrier prevents reflux of gastric contents into the oesophagus, while peristalsis helps to clear the reflux, to reduce exposure to the noxious components of the gastric juice. In this study, we found NERD and FH patients showed similar rates of hiatal hernia, and similar values of TBTT and CBTR; therefore, the two groups showed similarly impaired oesophageal clearance.

IEM, also known as oesophageal hypocontractility, is a manometric pattern characterised by ineffective swallows with poor bolus transit in the distal oesophagus. In the Chicago Classification (ver. 3.0), IEM is defined on Clouse plots using a DCI of <450 mmHg∙s∙cm, with > 50% ineffective swallows; IEM is highly prevalent in GERD patients [25, 26]. In our study, we found NERD patients presented a higher IEM rate. IEM is associated with the presence of abnormal acid reflux, as assessed by 24-h oesophageal pH-metry, regardless of the presence of defective LES, hiatal hernia, or oesophagitis [27]. However, the two groups in our study showed no statistically significant difference. Defects in the integrity of the peristaltic wave will lead to impaired bolus transit and prolonged oesophageal acid exposure [28]. William et al. [29] reported that longer breaks in the peristaltic wave predicted incomplete bolus clearance. Ribolsi et al. [30] reported that weak peristalsis with large breaks was associated with high acid exposure and delayed reflux clearance in the supine position in GERD patients. In the Chicago Classification (ver. 3.0), fragmented peristalsis (FP) is defined as DCI > 450 mmHg∙s∙cm, and break > 5 cm in the 20-mmHg isobaric contour, with >50% of ineffective swallows. In our study, NERD and RH patients showed similar FP rates.

Savarino et al. [31] reported that an increased number of weakly acidic reflux events and a high rate of proximal reflux are the main causes of symptoms in RH patients who are evaluated with MII/pH. Tamura et al. [6] reported total and proximal acid reflux events were significantly higher in NERD patients with abnormal acid exposure than in RH patients. In our study, MII/pH also showed that NERD patients presented more acid exposure time, bolus exposure time, and proximal and distal acid reflux events than RH patients. We also found that distal non-acid reflux events were more common in RH patients than in NERD patients.

Recently, impedance values for evaluation of oesophageal chemical clearance (PSPW index) and mucosal integrity (MNBI) have been proposed [13, 14]. After a reflux episode, oesophageal clearance is primarily achieved by secondary peristalsis, which removes around 90% of the reflux and is elicited by stretch receptors in the oesophageal lining (volume clearance); however, a neutral oesophageal pH is restored only after a voluntary swallow elicited by an oesophagosalivary reflex mediated through vagal afferents and delivery of salivary bicarbonate (chemical clearance) [32]. Impedance monitoring allows assessment of chemical clearance independently of volume clearance: a decrease in impedance originating in the upper oesophagus and reaching the lower oesophagus signals peristaltic transit of saliva and has been defined as a PSPW [13]. MNBI consists of the mean of three 10-min night-time periods, which accurately reflects the 6-h nocturnal bedtime period, is scarcely influenced by swallowing activity, and can reflect the reflux-induced impairment of mucosal integrity [14]. Analysis of impedance-pH data by calculating the PSPW index and the MNBI can increase the accuracy in patients with reflux disease compared with pH-only data [33]. Moreover, lower PSPW index and MNBI values have been found in erosive reflux disease than in NERD; both are significantly lower than those in patients with functional heartburn [34].

In this study, NERD patients had significantly lower PSPW index and MNBI values, greater acid exposure time and bolus exposure time, and more proximal and distal reflux events than RH patients. Impairment of chemical clearance, as indicated by a low PSPW index, implies prolonged contact of the oesophageal mucosa with acidic and weakly acidic refluxes [13]. A lower MNBI reflects impaired mucosal integrity [14].

Our study had some limitations. All subjects were recruited from two centres in one city using the Western normal range for MII/pH, and lacked a symptom severity score, which may have caused selection bias. The small number of patients limited the statistical power of the study. However, we are the first to compare oesophageal function tests between NERD and RH in Chinese patients.

## Conclusions

NERD and RH patients showed similar values on HRiM. NERD patients had greater acid exposure time, bolus exposure time, numbers of proximal and distal acid reflux events, and increased impairment of chemical clearance and mucosal integrity than RH patients. NERD and RH should be classified correctly by MII/pH to provide adequate relief from related symptoms.

## Abbreviations

DCI: Distal contractile integral
DEA: Distal oesophageal amplitude
EGJ: Oesophagogastric junction
GERD: Gastro-oesophageal reflux disease
HRiM: High-resolution manometry and impedance
IEM: Ineffective oesophageal motility
IRP: Integrated relaxation pressure
LES: Lower oesophageal sphincter
LESL: Length of lower oesophageal sphincter
LESP: Lower oesophageal sphincter pressure
LESRP: Lower oesophageal sphincter residual pressure
MII/pH: 24-h multi-channel intraluminal impedance and pH recording
NERD: Non-erosive reflux disease
RH: Reflux hypersensitivity
SAP: Symptom association probability
UESP: Upper oesophageal sphincter pressure

## References

[1] Vakil N, van Zanten SV, Kahrilas P, Dent J, Jones R (2006). Global Consensus Group. The Montreal definition and classification of gastroesophageal reflux disease: a global evidence-based consensus. Am J Gastroenterol.

[2] Winter JW, Heading RC (2008). The nonerosive reflux disease-gastroesophageal reflux disease controversy. Curr Opin Gastroenterol.

[3] Hachem C, Shaheen NJ (2016). Diagnosis and Management of Functional Heartburn. Am J Gastroenterol.

[4] Surdea Blaga T, Dumitrascu D, Galmiche JP (2013). Bruley des Varannes S. Functional heartburn: clinical characteristics and outcome. Eur J Gastroenterol Hepatol.

[5] Aziz Q, Fass R, Gyawali CP, Miwa H, Pandolfino JE, Zerbib F (2016). Functional esophageal Disorders. Gastroenterology.

[6] Tamura Y, Funaki Y, Izawa S, Iida A, Yamaguchi Y, Adachi K (2015). Pathophysiology of functional heartburn based on Rome III criteria in Japanese patients. World J Gastroenterol.

[7] Bredenoord AJ, Kahrilas PJ, Pandolfino JE, Schwizer W, Smout AJPM (2012). The International High Resolution Manometry Working Group: Chicago classification criteria of esophageal motility disorders defined in high resolution esophageal pressure topography. Neurogastroenterol Motil.

[8] Kahrilas PJ, Ghosh SK, Pandolfino JE (2008). Esophageal motility disorders in terms of pressure topography: the Chicago Classification. J Clin Gastroenterol.

[9] Kahrilas PJ, Bredenoord AJ, Fox M, Gyawali CP, Roman S, Smout AJ (2015). International High Resolution Manometry Working Group. The Chicago Classification of esophageal motility disorders, v3.0. Neurogastroenterol Motil.

[10] Shay S, Tutuian R, Sifrim D, Vela M, Wise J, Balaji N (2004). Twenty-Four hour ambulatory simultaneous impedance and pH monitoring : a multicenter report of normal values from 60 healthy volunteers. Am J Gastroenterol.

[11] Kushnir VM, Sathyamurthy A, Drapekin J, Gaddam S, Sayuk GS, Gyawali CP (2012). Assessment of concordance of symptom reflux association tests in ambulatory pH monitoring. Aliment Pharmacol Ther.

[12] Patel A, Sayuk GS, Gyawali CP (2015). Parameters on esophageal pH-impedance monitoring that predict outcomes of patients with gastroesophageal reflux disease. Clin Gastroenterol Hepatol.

[13] Frazzoni M, Manta R, Mirante VG, Conigliaro R, Frazzoni L, Melotti G (2013). Esophageal chemical clearance is impaired in gastro-esophageal reflux disease: a 24 h impedance-pH monitoring assessment. Neurogastroenterol Motil.

[14] Martinucci I, De Bortoli N, Savarino E, Piaggi P, Bellini M, Antonelli A (2014). Esophageal baseline impedance levels in patients with pathophysiological characteristics of functional heartburn. Neurogastroenterol Motil.

[15] Modlin IM, Hunt RH, Malfertheiner P, Moayyedi P, Quigley EM, Tytgat GN (2009). Diagnosis and management of non-erosive reflux disease--the Vevey NERD Consensus Group. Digestion.

[16] Dean BB, Gano AD, Knight K, Ofman JJ, Fass R (2004). Effectiveness of proton pump inhibitors in nonerosive reflux disease. Clin Gastroenterol Hepatol.

[17] Wu JC, Cheung CM, Wong VW, Sung JJ (2007). Distinct clinical characteristics between patients with nonerosive reflux disease and those with reflux esophagitis. Clin Gastroenterol Hepatol.

[18] Carlsson R, Dent J, Watts R, Riley S, Sheikh R, Hatlebakk J (1998). Gastrooesophageal reflux disease in primary care: an international study of different treatment strategies with omeprazole. International GORD Study Group. Eur J Gastroenterol Hepatol.

[19] Savarino E, Tutuian R, Zentilin P, Dulbecco P, Pohl D, Marabotto E (2010). Characteristics of reflux episodes and symptom association in patients with erosive esophagitis and nonerosive reflux disease: study using combined impedance-pH off therapy. Am J Gastroenterol.

[20] Rosaida MS, Goh KL (2004). Gastro-oesophageal reflux disease, reflux oesophagitis and non-erosive reflux disease in a multiracial Asian population: a prospective, endoscopy based study. Eur J Gastroenterol Hepatol.

[21] Bredenoord AJ, Weusten BL, Curvers WL, Timmer R, Smout AJ (2006). Determinants of perception of heartburn and regurgitation. Gut.

[22] Savarino E, Zentilin P, Savarino V (2013). NERD: an umbrella term including heterogeneous subpopulations. Nat Rev Gastroenterol Hepatol.

[23] Emerenziani S, Sifrim D, Habib FI, Ribolsi M, Guarino MP, Rizzi M (2008). Presence of gas in the refluxate enhances reflux perception in non-erosive patients with physiological acid exposure of the oesophagus. Gut.

[24] Emerenziani S, Ribolsi M, Sifrim D, Blondeau K, Cicala M (2009). Regional oesophageal sensitivity to acid and weakly acidic reflux in patients with non-erosive reflux disease. Neurogastroenterol Motil.

[25] Diener U, Patti MG, Molena D, Fisichella PM, Way LW (2001). Esophageal dysmotility and gastroesophageal reflux disease. J Gastrointest Surg.

[26] Savarino E, Gemignani L, Pohl D, Zentilin P, Dulbecco P, Assandri L (2011). Oesophageal motility and bolus transit abnormalities increase in parallel with the severity of gastro-oesophageal reflux disease. Aliment Pharmacol Ther.

[27] de Miranda Gomes PR, da Rosa AR P, Sakae T, Simic AP, Ricachenevsky Gurski R (2010). Correlation between pathological distal esophageal acid exposure and ineffective esophageal motility. Acta Chir Iugosl.

[28] Pandolfino JE, Roman S (2011). High resolution manometry: an atlas of esophageal motility disorders and findings of GERD using esophageal pressure topography. Thorac Surg Clin.

[29] William JB, Peter JK, Monika AK, Sudip KG, Albert M, John EP (2009). Esophageal pressure topography criteria indicative of incomplete bolus clearance: a study using high-resolution impedance manometry. Am J Gastroenterol.

[30] Ribolsi M, Balestrieri P, Emerenziani S, Guarino MP (2014). Cicala Michele. Weak peristalsis with large break is associated with high acid exposure and delayed reflux clearance in the supine position in GERD patients. Am J Gastroenterol.

[31] Savarino E, Zentilin P, Tutuian R, Pohl D, Gemignani L, Malesci A (2012). Impedance-pH reflux patterns can differentiate non-erosive reflux disease from functional heartburn patients. J Gastroenterol.

[32] Shafik A, El-Sibai O, Shafik AA (2005). Mostafa Rl. Effect of topical esophageal acidification on salivary secretion: identification of the mechanism of action. J Gastroenterol Hepatol.

[33] Frazzoni M, Savarino E, de Bortoli N, Martinucci I, Furnari M, Frazzoni L (2016). Analyses of the Post-reflux swallow-induced peristaltic wave index and nocturnal baseline impedance parameters increase the diagnostic yield of impedance-pH monitoring of patients with reflux disease. Clin Gastroenterol Hepatol.

[34] Frazzoni M, de Bortoli N, Frazzoni L, Tolone S, Furnari M, Martinucci I, at al. The added diagnostic value of postreflux swallow-induced peristaltic wave index and nocturnalbaseline impedance in refractory reflux disease studied with on-therapy impedance-pH monitoring. Neurogastroenterol Motil. 2017; 29(3). doi: 10.1111/nmo.12947. 10.1111/nmo.12947 27620303

